# The effects of touch-screen technology usage on hand skills among preschool children: a case-control study

**DOI:** 10.12688/f1000research.25753.1

**Published:** 2020-11-09

**Authors:** Ahmad Zamir Che Daud, Nurul Afiq'ah Aman, Chi-Wen Chien, Jenni Judd

**Affiliations:** 1 Centre of Occupational Therapy Studies, Universiti Teknologi MARA, Puncak Alam, Selangor, 42300, Malaysia; 2 Department of Rehabilitation Sciences, Hong Kong Polytechnic University, Hung Hom, Kowloon, Hong Kong; 3 School of Health, Medical and Applied Science|Centre for Indigenous Health Equity Research, Central Queensland University, Bundaberg, Queensland, Australia

**Keywords:** Child Behaviour, Child Development, Hand, Motor Skills, Screen Time

## Abstract

**Background:** Little is known on how time spent on touch-screen technology affects the hand skills development of preschool children. This study aimed to investigate the effects of touch-screen technology usage on hand skills among preschool children.

**Methods:** Case-control design was employed to compare the hand skills of children who were engaged in touch-screen technology. A total of 128 participants aged between five and six years old who attended preschool were recruited and divided into two groups: high usage touch-screen technology (HUTSTG) and, low usage touch-screen technology (LUTSTG). Children's Hand Skills ability Questionnaire (CHSQ) and Assessment of Children's Hand Skills (ACHS) were used to evaluate the children's hand skills.

**Results:** There were significant differences in the hand skills of preschool children between HUTSTG and LUTSTG. Results showed that preschool children in LUTSTG had better hand skills in all domains of CHSQ (p≤0.001) and ACHS (p<0.001) as compared to HUTSTG.

**Conclusion:** Frequent use of touch-screen technology might cause disadvantages to the development of hand skills among preschool children.

## Introduction

The proliferation of electronic technology in recent years has played a significant role in people's everyday life. As these technology usages become more crucial in everyday life, the prevalence of the possession of devices among teens has become very high in numbers and is escalating among younger children ^1^. Touch-screen technology usage among children had increased rapidly from 8%t to 40% in two years ^2^. Time spent using touch-screen technology has multiplied three times from 5 hours per day in 2011 to 15 hours per day in 2013 ^1^.

Instead of engaging and exploring their surroundings, children tend to spend most of their time playing and interacting with touch-screen technology ^3^. Children use touch-screen technology for various functions such as watching videos, playing games and listening to songs ^4^. Touch-screen technology has altered the way children play, interact and learn due to the interactive application of touch-screen technology that offers some entertainment and attractions ^5^. Interactive technology becomes an issue as motor development is dependent on children's motor experience ^6^. As children manipulate objects in their surroundings using their hands and fingers, it involves motor coordination, joint stability, muscle strength, visual perception and touch-ability. However, when using touch-screen technology, the involvement of motor coordination, muscle strength and dexterity are relatively low when compared to activities such as drawing, handwriting or playing with objects and toys ^7^. Touch-screen technology usage reduces the need to use hand skills such as grasping, in-hand manipulation and reaching as it only involves necessary fingers movements such as tapping, pressing, zooming and double-tapping ^8^.

Frequent engagement in touch-screen technology reduces children's chances to be involved in physical activities and may hinder the development of mature hand skills ^9^. One study reported that there was an improvement in manual dexterity, fine motor integration, and fine motor precision in typically developing preschool children who did not use any touch-screen technology for 24 weeks compared to the children who frequently engaged with touch-screen technology ^7^. This finding supports the assumption that frequent use of touch-screen technology without specific purposes, such as playing games and watching videos, might limit hand skill development of children.

However, the usage of this technology has been claimed to bring advantages, especially to those who learn kinesthetically through touch, movement and gesture ^10^. There is a positive correlation between touch-screen technology usage and hand skills ability ^11, 12^. A previous study reported that children who actively engaged in touch-screen technology developed hand skills ability earlier suggesting that the use of touch-screen technology may improve the efficiency of hand skills ^8^. Therefore the engagement with touch-screen technology could be considered as a healthy exercise for growing children ^4^. With the use of fingers to operate touch-screen technology, children have no risk of injury as compared to playing outside. Children would gain better knowledge of their hands and finger use, and become more efficient in a short period. Despite the positive association between touch-screen technology usage and hand skills, little is known on how the time spent using this technology may affect development of hand skills of preschool children. In the event where touch-screen technology has turned into an imperative method of play, analyzing its impact on the hand skills ability of children is exceptionally important. Therefore, this study explored the effects of time spent using touch-screen technology and hand skills among preschool children.

Children's age and gender, parents' level of education, their number of siblings, and family socioeconomic status need to also be considered as these factors that may affect children's hand skills other than touch-screen technology usage alone. In particular, family plays a significant role in early childhood development years. Older siblings might be a model for the development of motor skills in a younger child, and the number of siblings may have a correlation with hand skills of children ^13, 14^. Mother's educational level may have an association with a higher level of cognitive and higher earnings, which leads to better opportunities for children's development and exploration ^15^. Socioeconomic status has also been found to be a predictor to the performance of hand skills among children ^16, 17^. Children that had better socioeconomic status accomplished better hand skills as compared to children with low socioeconomic status due to early school years that allowed children to engage in various activities at school ^18^. There are a few studies that report the association between gender and hand skills. One study reported a likely low association between gender and hand skills ^19^. Girls accomplished better in manual dexterity skills, especially in paper-based and pencil-based activities as compared to boys ^20– 22^. Age, was also reported as a predictor for the development of hand skills and was more noticeable among younger children ^23, 24^. A recent study found that age plays a significant role in hand skill development ^25^. Since these personal and environmental factors might influence the hand skills of children, this study also aimed to examine the interaction of each element on hand skills.

The main research question this study addressed was: what are the effects of touch-screen technology usage on hand skills of pre-school children?

## Methods

### Study design and setting

A case-control study design was employed to investigate the hand skills between high usage touch-screen technology (HUTSTG) and low usage touch-screen technology (LUTSTG) groups. This observational study design allows the researchers to investigate the status of exposure of touch-screen technology without any interventions on hand skills ^26^. The study design was able to determine whether low or high exposure of touch-screen technology is associated with the outcomes (hand skills) ^27^. Demographic characteristics, hand skills ability and frequency of touch-screen technology usage data were collected from August to September 2019.

Participants were divided into two groups based on their touch-screen technology usage in accordance with Malaysian Dietary Guidelines, which recommends screen time among preschool children should be less than two hours per day ^28^. Children who used touch-screen technology more than two hours per day were allocated to the HUTSTG group (case), and if they did not, they were assigned to the LUTSTG group (control). The guideline for screen time was developed to promote natural development and a life balance between sleep, sedentary behavior and physical activity among children in a 24-hour period ^28, 29^. Touch-screen technology usage included all screen-based activities using a tablet or smart phone such as playing games, using mobile applications and watching videos. Touch-screen technology usage was reported by parents or caregivers in the number of minutes. Recruitment of participants was from the Ministry of Education (MOE) preschools in Southern Region of Malaysia.

### Participants

Purposive sampling was used to recruit the participants. Participants were then randomly selected by using computer generated random number in order to reduce the possibility of the human bias in cases selection. Inclusion criteria for HUTSTG group were typically developing children: (i) aged 5–6 years old; (ii) attending the MOE preschool; (iii) engaging in touch-screen technology (either phone or tablet) for more than 120 minutes per day, according to parent-report; and (iv) parents are also able to participate in this study to complete the CHSQ. While for LUTSTG, the inclusion criteria were similar for HUTSTG except than the children engaging in touch-screen technology (either phone or tablet) for less than 120 minutes per day according to the parent report. Preschool children that had been diagnosed with diseases or disorders associated with developmental delays based on parents' report were excluded from this study for both groups.

Sample size calculation was calculated using the G*Power 3.1 software, with power set at 0.80 to detect the medium-effect size (d = 0.50) and α = 0.05 with allocation ratio N2/N1 of 1. Therefore, a total of 128 participants with 64 children in each group were recruited to achieve adequate power.

### Data collection procedures

Initially, parents of preschool children were asked to complete the Children's Hand Skills ability Questionnaire (CHSQ) that was used to evaluate the uses of the hands by the children aged 2–12 years old while performing activities of the daily life from the perspectives of the parents or the caregivers ^30^. This CHSQ assessment comprises of 21 activities of the hand skills that are divided into three domains of leisure and play (8 activities), school/education (8 activities), and Activities of Daily Living (ADL) (6 activities). Likert scale with three-level was used in the CHSQ that are; 1 (extremely difficult), 2 (difficult) and 3 (no difficulty) indicating the difficulty level while performing the 21 activities. The "not applicable" was marked if the child does not perform the activity in the last three months. CHSQ assessment can be used as a companion assessment to know the range of difficulties in performing hand skill activities before evaluating the children's hand skills using Assessment of Children's Hand Skills (ACHS) ^31^. CHSQ is reported to have a sufficient person-response validity, and satisfactory internal and external construct validity to capture children's' manual ability in the domain of ADL, leisure and play; and school/education ^30^. Average scores for each domain in CHSQ were calculated to determine the difficulty level of the child while engaging in leisure and play, school/education and ADL activities.

A performance-based observation was then conducted with all participants using ACHS. This naturalistic observational assessment was used in this present study to assess the actual performance of the child's hand skills in a real-life context when engaging in several types of daily activities in everyday settings ^32^. This standardized assessment comprised of 5 categories of hand skills items; (1) hand skills without interacting with objects (manual gesture, body contact); (2) "arm-hand" use object-related hand skills (reaching, turning, carrying, throwing, catching, moving, stabilizing); (3) "adaptive skilled hand use" object-related hand skills (grasping, holding, in-hand manipulating, releasing, isolated finger movements); (4) "bimanual use" object-related hand skills (transferring, using both hands simultaneously, using both hands cooperatively); and (5) general quality of hand skills (accuracy, pace, movement quality). These hand skills items for each activity are rated on a 6-point rating scales; a score of 6 indicates very effective hand skill performance, while a score of 1 indicates very ineffective hand skill performance. Two or three activities that were scored 1 (extremely difficult) or 2 (difficult) by parents in the CHSQ were observed. Observations and scoring were done while children performed the selected activities for 20–30 minutes at their preschools. Evidence for the content, construct, discriminant and convergent validity of ACHS were demonstrated, indicating that this assessment can be used with confidence to measure children's real-life hand skill performance ^31, 33, 34^. ACHS is also reported to have acceptable intra-rater, inter-rater and test-retest reliability to quantify children's hand skills use ^32, 33^. Composite scores of ACHS were computed to determine the overall hand skills performance of preschool children while doing all the selected activities.

### Data analysis

Chi-square and Fisher‘s exact test was conducted to determine whether differences in demographic characteristics of participants existed between HUTSTG and LUTSTG groups. Independent T-test was used in order to investigate any significant differences in hand skills ability among preschool children between HUTSTG and LUTSTG groups. Two-way analysis of variance (ANOVA) was conducted in order to explore the effects and interactions of personal and environmental factors (age of the preschool children, gender of the preschool children, number of siblings, household income status, mother‘s age, mother‘s educational level) on hand skills ability between HUTSTG and LUTSTG groups. Lastly, Pearson’s correlation coefficients was computed in order to investigate the relationship between the children‘s hand skills ability based on parents‘ reported questionnaire, CHSQ and performance-based observation, ACHS. Missing data was treated using expectation maximization.

### Ethical considerations

The ethics review committee of Universiti Teknologi MARA (UiTM) (REC/223/19) approved the study. Permissions from the Ministry of Education (KPM.600-3/2/3-eras-3447) and Johor State Education Department (JPNJ.PP.600-1/1/2Jld.2-42) were also obtained as this study involved the preschool children under the MOE. Before the preschool children participated in the study, the procedures were explained to the parents, and their written consent for their child and themselves to participate was given.

## Results

### Participant's demographic characteristics

The HUTSTG and LUTSTG groups each consisted of 64 preschool children in which the gender (male and female), and age (five years and six years) were balanced between the groups ( Table 1). There were no significant differences in all demographic characteristics of the participants between HUTSTG and LUTSTG groups.

**Table 1.** Comparison of demographic characteristics of the participants between groups.

Characteristics	LUTSTG n (%)	HUTSTG n (%)	P-value
***Gender***			
Male	32 (50.0)	32 (50.0)	0.570 ^a^
Female	32 (50.0)	32 (50.0)	
***Age (years)***			
5	32 (50.0)	32 (50.0)	0.570 ^a^
6	32 (50.0)	32 (50.0)	
***Number of siblings***			
1 – 2	22 (34.4)	21 (32.8)	0.268 ^b^
3 – 4	37 (57.8)	32 (50.0)	
More than 4	5 (7.8)	11 (17.2)	
***Mother's age (years)***			
21 – 30	7 (10.9)	7 (10.9)	0.482 ^b^
31 – 40	49 (76.6)	44 (68.8)	
More than 40	8 (12.5)	13 (20.3)	
***Mother's educational level***			
Secondary School	38 (59.4)	43 (67.2)	0.232 ^a^
Higher Education	26 (40.6)	21 (32.8)	
***Household income***			
Less than RM 3,000	39 (60.9)	48 (75.0)	0.065 ^a^
More than RM 3,000	25 (39.1)	16 (25.0)	

^a^Fisher's Exact Test; ^b^Chi-Square

### Touch-screen technology usage and hand skills

Results showed that there were significant differences for all domains in CHSQ between LUTSTG and HUTSTG groups. Table 2 shows the results of independent t-tests for both groups using the average scores of each domain in CHSQ. There was also a significant difference of the hand skill performance of ACHS between LUTSTG (M = 2.702, SD = 1.808) and HUTSTG (M = 0.956, SD = 1.266) groups with t (126) = 6.329, p = 0.000.

**Table 2.** The effects of touch-screen technology usage on hand skills.

Hand skills		Mean ± SD		Mean diff. (95% CI)	T-stats (df)	P-value
		LUTSTG (n=64)	HUTSTG (n=64)			
Performance based evaluation (ACHS)		2.702 ± 1.808	0.956 ± 1.266	1.746 (1.200, 2.292)	6.329 (126)	< 0.001
Parent's reported evaluation (CHSQ)	Play/leisure domain	2.908 ± 0.176	2.788 ± 0.199	0.120 (0.054, 0.185)	3.616 (126)	< 0.001
	School/education domain	2.929 ± 0.170	2.784 ± 0.206	0.145 (0.079, 0.211)	4.352 (126)	< 0.001
	Activities of daily living domain	2.990 ± 0.041	2.945 ± 0.100	0.045 (0.018, 0.072)	3.326 (126)	0.001

### Effects of personal and environmental factors on hand skills based on ACHS

Two-way ANOVA was conducted to examine the factors that may affect hand skills based on the performance-based evaluation, ACHS. Table 3 shows that there were non-significant interactions between touch-screen technology usage and all the factors (gender, age, number of siblings, mother's age, mother's educational level and household income). The effects of touch-screen technology usage on hand skills (ACHS) were not influenced by those factors, and the hand skills of preschool children were influenced solely by touch-screen technology usage.

**Table 3.** Interaction effects of personal and environmental factors on hand skills among preschool children (N=128).

Factors	ACHS		CHSQ					
			Play and Leisure		School/Education		Activities of Daily Living	
	F-value(df)	P-value	F-value(df)	P-value	F-value(df)	P-value	F-value(df)	P-value
**Gender of preschool children** Touch-screen technology usage Gender of preschool children Touch-screen technology usage X Gender	39.591 (1, 124) 0.159 (1, 124) 0.388 (1, 124)	0.000 0.691 0.535	12.922 (1, 124) 0.033 (1, 124) 0.514 (1, 124)	0.000 0.855 0.475	18.860 (1, 124) 1.480 (1, 124) 0.001 (1, 124)	0.000 0.226 0.978	11.080 (1, 124) 0.323 (1, 124) 1.841 (1, 124)	0.001 0.571 0.177
**Age of preschool children** Touch-screen technology usage Age of preschool children Touch-screen technology usage X Age	41.185 (1, 124) 5.249 (1, 124) 0.312 (1, 124)	0.000 0.024 0.578	12.999 (1, 124) 1.283 (1, 124) 0.003 (1, 124)	0.000 0.259 0.959	19.132 (1, 124) 3.276 (1, 124) 0.015 (1, 124)	0.000 0.073 0.903	11.080 (1, 124) 0.323 (1, 124) 1.841 (1, 124)	0.001 0.571 0.177
**No. of siblings** Touch-screen technology usage No. of sibling Touch-screen technology usage X No. of siblings	14.465 (1, 122) 0.591 (2, 122) 2.692 (2, 122)	0.000 0.555 0.072	10.595 (1, 122) 0.360 (2, 122) 1.126 (2, 122)	0.001 0.698 0.328	14.420 (1, 122) 0.840 (2, 122) 0.278 (2, 122)	0.000 0.434 0.758	10.207 (1, 122) 1.502 (2, 122) 2.393 (2, 122)	0.002 0.227 0.096
**Mother's age** Touch-screen technology usage Mother's age Touch-screen technology usage X Mother's age	12.686 (1, 122) 0.368 (2, 122) 1.219 (2, 122)	0.001 0.693 0.299	9.289 (1, 122) 0.034 (2, 122) 0.262 (2, 122)	0.003 0.967 0.770	7.945 (1, 122) 0.802 (2, 122) 1.447 (2, 122)	0.006 0.451 0.239	9.932 (1, 122) 0.612 (2, 122) 0.973 (2, 122)	0.002 0.544 0.381
**Mother's educational level** Touch-screen technology usage Mother's educational level Touch-screen technology usage X Educational level	44.270 (1, 124) 0.110 (1, 124) 3.692 (1, 124)	0.000 0.741 0.057	9.778 (1, 124) 3.117 (1, 124) 0.748 (1, 124)	0.002 0.080 0.389	15.489 (1, 124) 5.167 (1, 124) 0.199 (1, 124)	0.000 0.025 0.656	8.750 (1, 124) 1.075 (1, 124) 0.300 (1, 124)	0.004 0.302 0.585
**Household income** Touch-screen technology usage Household income Touch-screen technology usage X Household income	42.362 (1, 124 0.034 (1, 124) 3.726 (1, 124)	0.000 0.855 0.056	7.595 (1, 124) 4.923 (1, 124) 0.633 (1, 124)	0.007 0.028 0.428	15.531 (1, 124) 3.234 (1, 124) 0.251 (1, 124)	0.000 0.075 0.618	5.915 (1, 124) 4.707 (1, 124) 0.941 (1, 124)	0.016 0.032 0.334

### Effects of personal and environmental factors on hand skills based on CHSQ

Two-way ANOVA was also conducted to examine the factors that may affect hand skills based on parent's reported questionnaire, CHSQ. Table 3 demonstrates that there were no significant interactions between touch-screen technology usages. All factors such as gender, age, number of siblings, mother's age, mother's educational level, and household income did not influence hand skills; in contrast, the effects of touch-screen technology usage on hand skills for all domains in the CHSQ (Play and Leisure, School/Education and ADL) did.

### Correlation of parents' reported questionnaire (CHSQ) and performance-based evaluation (ACHS)

CHSQ represented the parents' reported hand skills and ACHS represented performance-based observation of hand skills. The correlation between these two measures was studied. There was a non-significant correlation between ACHS score and the play/leisure domain of CHSQ, r = 0.082, p = 0.358. However, there was a very weak positive correlation between ACHS score and school/education domain of CHSQ (r=0.231, p=0.009). Similarly, there was a weak positive correlation, r=0.187, p=0.035 ( Table 4) between ACHS score and activities of daily living (ADL) domain.

**Table 4.** Correlation between hand skills of parent-reported and performance-based evaluation (N=128).

Hand skills		Performance-based evaluation (ACHS)	
		Pearson Correlation (r)	P-value
Parents' reported evaluation (CHSQ)	Play and leisure domain	0.082	0.358
	School/education domain	0.231	0.009
	Activities of daily living (ADL) domain	0.187	0.035

## Discussion

This study aimed to examine the effects of touch-screen technology usage on hand skills ability of preschool children and proposed to explore the factors that might influence the hand skills of the children. Both parents' reported questionnaire (CHSQ) and performance-based evaluation (ACHS) showed significantly better hand skills in the LUTSTG group compared with the HUTSTG group. The results suggest that children that are engaged with touch-screen technology for more than two hours per day have less adequate hand skills as compared to children who use touch-screen technology for less than two hours per day. The results were aligned with the assumption that frequent usage of touch-screen technology might limit the development of hand skills among preschool children ^7^. As presumed by 35, traditional games such as puzzles, board games and construction blocks have been replaced by the use of touch-screen technology. Movements required when using touch-screen technology are different from those involved when playing traditional games, doing most activities of daily living and completing school tasks, and reduces the children's experiences in manipulating and handling objects in real life ^3, 9^, which may explain why children in the LUTSTG group have better hand skills.

The social context in which children live and are being raised, including the socioeconomic status of the family, mother's educational level and the number of siblings influenced the development of children ^13– 15, 17, 18^. Our study demonstrated no significant interaction effects between all personal and environmental factors (such as gender, age, number of siblings, mother's age, mother's educational level and household income) on hand skills for both HUTSTG and LUTSTG groups. Touch screen usage alone influenced the hand skills of preschool children in this study.

The parent-reported evaluation should be complemented alongside a real-life observational evaluation to elucidate the most precise evaluation ^32^. In this study, data from parents' reported questionnaire (CHSQ) was correlated with the data from the performance-based evaluation (ACHS). This study found that there was a positive correlation between the school/education and activities of daily living (ADL) domain of CHSQ and ACHS. It showed that school activities (writing, copying, coloring and drawing) and ADL activities (drinking, eating and washing hands) are the most common activities done in school and home environment. Parents' reported questionnaire on their children's hand skills (i.e., the results of CHSQ) is in agreement with the performance-based evaluation while children are engaged in school tasks and ADL activities in the preschool. However, the correlations were weak, and to evaluate the hand skills of children it is better to have a performance-based evaluation rather than parents' reported evaluation. For the leisure domain of CHSQ, there is no correlation between the domain and the ACHS score and showed a contradiction between the leisure activities observed by parents in the home environment with the performance-based evaluation done in the school environment. School is not a familiar place for children to frequently participate in leisure and play activities such as playing blocks, card games and stringing beads.

There were a few limitations found in this study. First, this study relied on parents' reports for the time spent by their children using touch-screen technology. Second, this study did not take into consideration the age of children when they first started using touch-screen technology. Early use of touch-screen technology might have effects on the hand skills of children. Thirdly, this study did not investigate other areas of development that might relate to the impact of touch-screen technology usage, for instance, the development of visual perception, language and cognitive functions of children. Use of touch-screen technology might be useful for improving visual perceptions, language or cognitive functions of children. Still, it showed otherwise in the hand skills of children.

Future study needs to obtain further information on children's use of touch-screen technology not just from parents but also their caregivers in their childcare centres. Future study also warrants the effects of touch-screen technology usage on the development of visual perceptions and cognitive functions.

## Conclusion

Positive and negative effects of touch-screen technology have been debated. Many previous studies have investigated the effects of touch-screen technology on several aspects of childhood development. The advantages may sometimes overshadow the disadvantages of this technology, however, they must be considered. Results of this study suggest that there are differences in hand skills development between HUTSTG and LUTSTG groups and those children with frequent use of touch-screen technology may have difficulties with their hand skills when performing daily life activities.

## Data availability

### Underlying data

The underlying data related to this article has been previously published in a data article by the authors: https://doi.org/10.1016/j.dib.2020.106358 ^36^. The data article contains the following data:

1) Raw data for analysis results of the effects of touch-screen technology usage on hand skills.

- Spreadsheet (16kb):

2) Raw scores and the calculated average for CHSQ assessment.

- Spreadsheet (41kb):

3) Raw scores for each item of ACHS assessment.

- Spreadsheet (34kb):

4) Computed composite scores of ACHS assessment.

- Spreadsheet (24kb):

Data are available under the terms of the Creative Commons Attribution 4.0 International license (CC-BY 4.0).
