# Supplementary material for: The effects of touch-screen technology usage on hand skills among preschool children: a case-control study
Source: F1000Res. 2020 Nov 9;9:1306. [Version 1] doi: 10.12688/f1000research.25753.1 (PMC8666989; doi:10.12688/f1000research.25753.1)
Supplement: Peer review history

# Peer review: Reviewer response for version 1

**DOI:** 10.5256/f1000research.28422.r100534

**Published:** 2021-12-09

**Authors:** Nerdy Nerdy

**Affiliations:** 1 Department of Pharmacy, Faculty of Pharmacy, Institut Kesehatan Deli Husada Deli Tua, Deli Tua Timur, Deli Tua, Deli Serdang, Sumatera Utara, Indonesia

- **Introduction:**
  - Adequate to describe the introduction, but please also be fair by stating acceptable negative side of gadget to children.
  - Please clearly state the novelty of the research.
- **Methodology: **Not enough to describe how to correlate the hand skill with the usage.
- **Results and Discussion:** Have been well enough to present the data and adequate literature to support the results.
- **Suggestion:** Please attached the similarity check report.

Is the work clearly and accurately presented and does it cite the current literature?

Yes

If applicable, is the statistical analysis and its interpretation appropriate?

Yes

Are all the source data underlying the results available to ensure full reproducibility?

Yes

Is the study design appropriate and is the work technically sound?

Partly

Are the conclusions drawn adequately supported by the results?

Yes

Are sufficient details of methods and analysis provided to allow replication by others?

Partly

Reviewer Expertise:

Pharmacology, Medicinal Chemistry, Pharmaceutical Chemistry, Pharmaceutical Science, Social Sciences, Health Science, Public Health

I confirm that I have read this submission and believe that I have an appropriate level of expertise to confirm that it is of an acceptable scientific standard.

# Peer review: Reviewer response for version 1

**DOI:** 10.5256/f1000research.28422.r74574

**Published:** 2020-12-15

**Authors:** Kounosuke Tomori

**Affiliations:** 1 Department of Occupational Therapy, School of Health Science, Tokyo University of Technology, Tokyo, Japan

The effects of touch-screen technology usage on hand skills among preschool children: a case-control study

**Abstract:**

 Background: 

- “Little is known on how time spent on touch-screen technology affects the hand skills development of preschool children.” You have frequently cited the literature that the negative effect of touch-screen technology and you have a hypothesis in the manuscript. So, I think you should show it in the background (I have been thought the group in high-intensity use the technology has good hand skills until reading the result!). 

**Introduction:**

- Advancing technology is very fast. In the first paragraph, you showed the literature to spend time using the touch-screen tool. But this data looks old a little bit. Please update this. (if possible) 
- The comprehensive literature review is good, but it is unclear what focus on your study. The sentence of research interesting & novelty needs to be added to the introduction section. What is the difference between your study from previous studies?

**Method:**

- Does the difference between HUTSTG and LUTSTG groups spend time using the technology devise over 120 min or not?
- If possible, please explain how to decide the effect size (d=0.5). 
- Who and how did data collection for CHSQ and ACHS? 
- Please explain the interpretation of the score of CHSQ and ACHS. Is a high score better or not?

Data analysis:

- You conducted the data analysis using two-way ANOVA. This analysis is not wrong, but still unclear what factor is effective in the result. Multivariate analysis is frequently applied to the case-control design. If the Logistic regression analysis is applied, the result shows an odds ratio, and the factors are comparable. Please re-consider the data analysis, if you agree with this.

**Discussion:**

- I think the discussion is well written. It may be more interesting if the data regarding traditional play or daily activities which affect to development of hand skill is collected. It is still unclear what factor was decreased instead of using the time the touch-screen device. 

Is the work clearly and accurately presented and does it cite the current literature?

Yes

If applicable, is the statistical analysis and its interpretation appropriate?

Partly

Are all the source data underlying the results available to ensure full reproducibility?

Yes

Is the study design appropriate and is the work technically sound?

Yes

Are the conclusions drawn adequately supported by the results?

Yes

Are sufficient details of methods and analysis provided to allow replication by others?

Yes

Reviewer Expertise:

NA

I confirm that I have read this submission and believe that I have an appropriate level of expertise to confirm that it is of an acceptable scientific standard.
